# New perspectives on fetal and neonatal alloimmune thrombocytopenia for obstetricians

**DOI:** 10.3389/fimmu.2026.1783176

**Published:** 2026-04-01

**Authors:** Yi Zhao, Peiyan Liu, Lixin Chen, Chinwe Oluchi-Amaka Ibeh, Joanne Kwak-Kim, Feng Guo, Xiuhua Yang

**Affiliations:** 1 Department of Obstetrics, The First Hospital of China Medical University, Shenyang, China; 2 Reproductive Medicine and Immunology, Obstetrics and Gynecology, Clinical Sciences Department, Chicago Medical School, Rosalind Franklin University of Medicine and Science, North Chicago, IL, United States; 3 Clinical Immunology Laboratory, Foundational Sciences and Humanities, Microbiology and Immunology, Chicago Medical School, Rosalind Franklin University of Medicine and Science, North Chicago, IL, United States; 4 Department of Emergency Medicine, Shengjing Hospital of China Medical University, Shenyang, China

**Keywords:** fetal and neonatal alloimmune thrombocytopenia, fetus, immune, platelet, pregnancy

## Abstract

Fetal and neonatal alloimmune thrombocytopenia (FNAIT) is a serious disorder that arises when a mother produces alloantibodies against specific human platelet antigens (HPAs) expressed on fetal platelets. These maternal alloantibodies cross the placenta and destroy fetal platelets, significantly increasing the risk of fetal or neonatal intracranial hemorrhage (ICH). Indeed, FNAIT is the leading cause of isolated severe thrombocytopenia in otherwise healthy neonates. In this review, we summarize recent advances to provide obstetricians and maternal-fetal medicine specialists with an updated overview of FNAIT, encompassing its pathophysiology, clinical features, diagnostic strategies, antenatal management, delivery planning, and postnatal care. Although the prophylactic interventions remain at an early stage, HPA histo-incompatibility prescreening is already available at a suitable cost, and the development of NAITgam, a targeted immunoglobulin preparation, represents a promising advance. While the widespread implementation of screening and prophylaxis may take years, such measures have the potential to significantly reduce the incidence and severity of FNAIT. Currently, the antenatal administration of intravenous immunoglobulin (IVIg), with or without corticosteroids, remains the safest and most effective treatment for high-risk pregnancies. Looking ahead, animal model data continue to provide valuable insights that may inform the development of novel preventive and therapeutic strategies. Ultimately, the implementation of a national screening program could prevent severe complications and help mitigate the long-term societal and healthcare burden of FNAIT.

## 1 Introduction

Fetal and neonatal alloimmune thrombocytopenia (FNAIT) is an alloimmune condition that occurs when a mother generates alloantibodies against histo-incompatible human platelet antigens (HPAs) existing on the platelets of her fetus. These maternal alloantibodies can destroy fetal platelets and impact vascular integrity and angiogenesis, thereby increasing the risk of fetal intracranial hemorrhage (ICH) (1–3). FNAIT constitutes the main cause of isolated severe thrombocytopenia in healthy term newborn (4). If the platelet count of a newborn is below 100×10^3^/μL at the time of birth or within 7 days after birth with symptoms of ICH, FNAIT can be diagnosed if laboratory investigation (maternal platelet antibody testing, paternal crossmatch and genotyping) supports the diagnosis (5). About 80% of Caucasian women with FNAIT have platelets expressing only HPA-1b (HPA-1a negative) and give birth to HPA-1a fetuses. In addition to HPA-1a mismatch, there are 37 types of platelet antigen incompatibilities that can lead to FNAIT; however, symptoms are often milder (6). Among them, anti-HPA-5b has been the second most prevalent antibody implicated in FNAIT (7).

Earlier studies have identified risk factors involved in the immunization process against HPA in pregnant women. In the case of certain antigens like HPA-1a, the presentation of the HPA-1a epitope on placental tissue appears to be particularly significant (8). Additionally, the presence of the MHC class II allele HLA-DRB3^*^01:01 in HPA-1a negative women can enhance the immune response against HPA-1a, as it facilitates efficient presentation of HPA-1a peptides (9, 10). In Asian populations, the HPA-1a and HPA-1b genetic variants are found to be extremely uncommon. Instead, the HPA-4 system is the most common type found in these groups (11). According to a Japanese study, the frequencies of the HPA-4b and HPA-5b polymorphisms were reported to be around 26% and 10%, respectively (12). Furthermore, previous occurrence of FNAIT or ICH is also a high-risk factor for FNAIT (13, 14).

The prevalence of FNAIT differs across various ethnic groups, largely influenced by the specific HPA type inherited by the fetus. Among individuals of Caucasian ancestry in North America and Europe, for example, the general occurrence of FNAIT is roughly 0.15% (15). Clinical manifestations range from petechiae to ICH in affected babies. The most serious complication of FNAIT is ICH, with an incidence rate of lower than one in 1,000. ICH often occurs before 28 weeks of gestation and even affects the first child (16, 17). ICH may result in perinatal death or lifelong neurological sequelae (18). Furthermore, FNAIT also affects mothers. More than half of FNAIT women experience psychological problems for more than 6 weeks, including anxiety, depression, and sleep disorders (19). Due to FNAIT being a rare disease, it is challenging to determine its impact on the general public (17).

Applying intravenous immunoglobulin (IVIg) to pregnant women with FNAIT during pregnancy may prevent ICH caused by FNAIT (20). However, due to the lack of well-designed, large-scale, randomized controlled trials (RCTs), ideal prenatal and postpartum management has not been established (20, 21). In this manuscript, we reviewed the literature on FNAIT, providing up-to-date advances and evidence-based insights into etiologies, clinical presentation, diagnostic strategies, antenatal management, optimal timing and mode of delivery, as well as postnatal care of affected newborns. Our goal is to mitigate adverse pregnancy outcomes associated with this condition, thereby reducing the associated socioeconomic and healthcare burden on society.

## 2 Etiology

### 2.1 Human platelet antigens

Typically, HPAs can be divided into high- and low-frequency antigens. Twelve high-frequency HPAs are grouped into six biallelic clusters, namely HPA-1, 2, 3, 4, 5, and 15. Based on published reports (12, 22–24), these high-frequency HPAs and their frequencies in various races are shown in **Table 1**. HPAs are polymorphic epitopes on five different cell-surface platelet glycoproteins (GP). These glycoproteins have a significant function in platelet adhesion, aggregation, and the formation of hemostatic plugs (25). The glycoprotein that has the majority of HPAs is glycoprotein IIIa (known as CD61), which is the β3 subunit of the integrin αIIbβ3 complex (26). The HPA-1 antigen is located on the β3 integrin subunit, defined by a Leu33Pro polymorphism within the Plexin-Semaphorin-Integrin (PSI) domain. The integrin αIIbβ3 complex, also known as the fibrinogen receptor, is expressed on platelets and megakaryocytes, and the β3 subunit of this receptor forms the integrin αVβ3 complex (fibronectin receptor), which is expressed on angiogenic endothelial cells and invasive trophoblast cells (27).

**Table 1:** High-frequency human platelet antigens (HPAs) and their frequencies in various races.

HPA name	Gene frequency					
	Caucasian (%)	African (Benin) (%)		Chinese (%)	Indian (%)	Japanese (%)
HPA-1a	85		90	100	88.4	99.8
HPA-1b	15		10	0	11.7	0.2
HPA-2a	93		71	95	94.1	86
HPA-2b	7		29	5	5.9	14
HPA-3a	61		68	59.5	65.3	54
HPA-3b	39		32	40.5	34.7	46
HPA-4a	>99		100	99.5	99.9	96.8
HPA-4b	<1		0	0.5	0.1	0.9
HPA-5a	89		82	98.6	92.3	94.5
HPA-5b	11		18	0.4	7.7	5.5
HPA-15a	51		65	53	58.2	–
HPA-15b	49		35	47	41.8	–

“-”, not reported.

The fetal megakaryocytes appear in the lung and liver of fetuses at the 12th week of pregnancy, and the platelet count of a normal fetus reaches adult levels no later than 18 weeks (28). Maternal IgG alloantibodies produced in the previous pregnancy can be detected in fetal blood from 6 weeks of gestation (29) and gradually increase with advancing gestational age (30). Therefore, fetal thrombocytopenia caused by maternal alloantibodies can occur in early pregnancy, and preconception evaluation of maternal platelet alloantibodies and treatment should be considered for women who were already sensitized or at high risk of developing FNAIT (31, 32).

### 2.2 Immune response of the mother and the fetus

**Figure 1:**
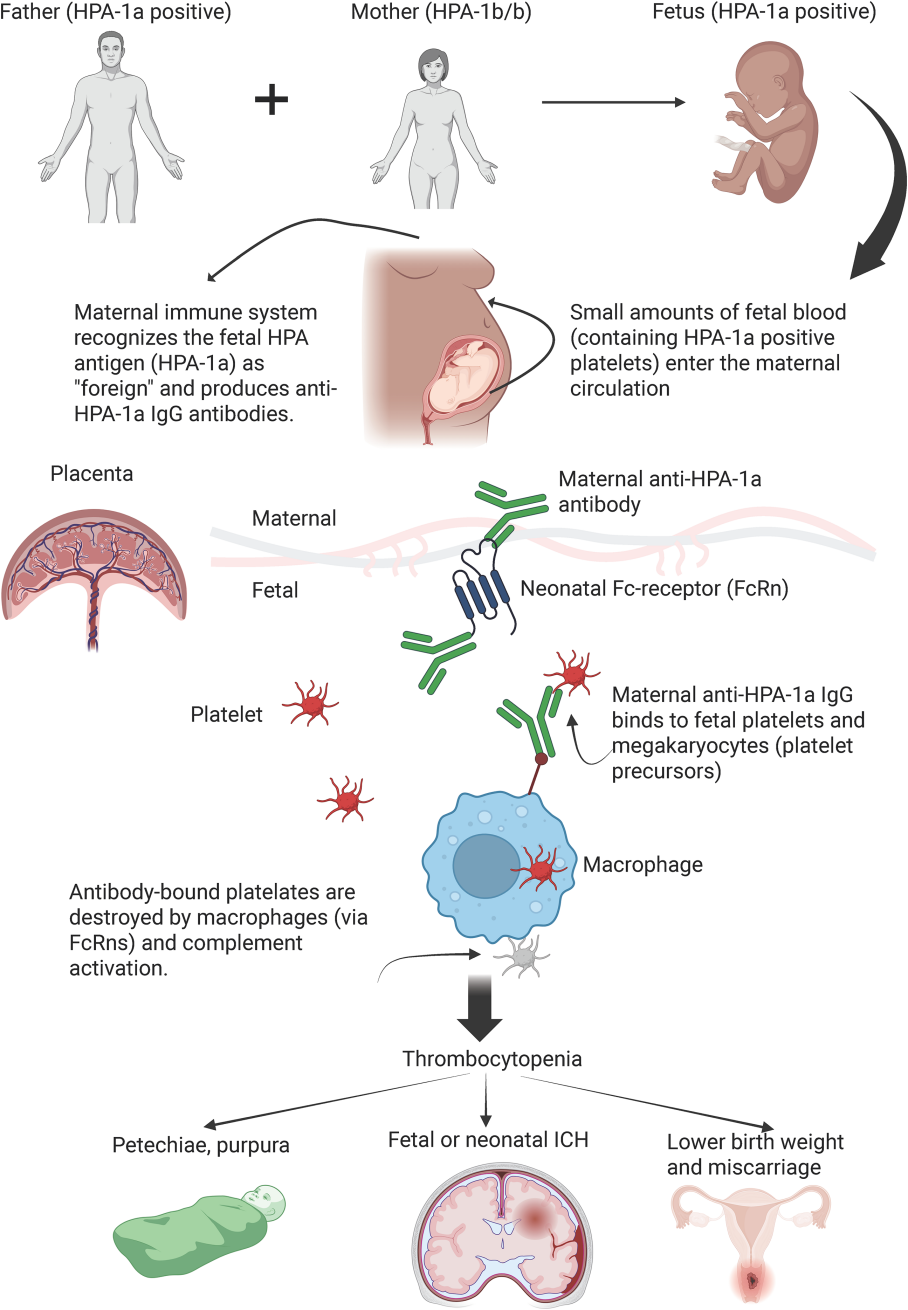
The pathogenesis of FNAIT. ICH, intracranial hemorrhage. This figure was generated by BioRender.

Exposure to paternally derived HPA can occur under both physiological and pathological conditions. Fetal blood cells may enter the maternal circulation spontaneously throughout gestation, but most commonly at the time of delivery. Additional exposure may occur following invasive perinatal procedures or abdominal trauma. Genetic susceptibility plays a critical role in the development of alloimmunization. Specifically, women carrying the HLA DRB3^*^01:01 allele have an approximately 25-fold increased risk of developing anti-HPA-1a antibodies compared to non-carriers (33). Among patients already immunized against HLA-1a, approximately 90% are HLA DRB3^*^01:01 positive (33). Furthermore, HPA-1a-immunized women who are HLA DRB3^*^01:01 tend to produce significantly higher levels of anti-HPA-1a antibodies (34). Maternal IgG-anti-HPA antibodies can cross the placenta via neonatal Fc receptors (FcRn), leading to fetal platelet destruction. The proposed pathophysiological mechanism of FNAIT is illustrated in **Figure 1**.

Bacterial or viral infections may enhance maternal immune responses to fetal platelet antigens (35). Although infections are known to contribute to the pathogenesis of idiopathic thrombocytopenic purpura (ITP), their role in the severity of FNAIT has not been fully elucidated. In a murine model study, platelets from wild-type mice were transfused into β3 or GPIbα knockout mice (β3^-/-^ and GPIbα^-/-^) to assess the immunogenicity of specific platelet antigens (35). Their results demonstrated that GPIbα is inherently less immunogenic than β3 integrin, which may explain why FNAIT, caused by anti-GPIbα, is less common than ITP. To simulate viral and bacterial infections, polyinosinic: polycytidylic acid (Poly I:C) and lipopolysaccharide (LPS) were administered intraperitoneally to the mice. Co-administration of Poly I:C or LPS significantly amplified the immune response against both antigens, particularly to GPIbα, and was associated with worse pregnancy outcomes, including fetal loss. These findings raise the possibility that maternal infections may exacerbate FNAIT by heightening alloimmune responses against fetal platelet antigens. In humans, 75-95% of FNAIT cases are characterized by anti-β3 integrin antibodies (36). Similarly, most cases of ITP are also caused by anti-β3 integrin antibodies. Interestingly, 20-40% of ITP patients are caused by anti-GPIbα antibodies (37), but patients with FNAIT mediated by anti-GPIbα antibodies are rare (<1%) (38, 39).

### 2.3 Anti-HPA-1a glycosylation

Researchers investigated sera from 48 females who were sensitized to HPA-1a and investigated variations in the glycosylation at Asn297 in the IgG-Fc, which affects binding to phagocyte IgG-Fc receptors (FcγR) (40). In women with FNAIT, core fucosylation of anti-HPA-1a-specific IgG1 was significantly reduced (40). Importantly, the degree of anti-HPA-1a fucosylation was negatively correlated with the severity of FNAIT, although the data were widely dispersed without a distinct cut-off (40). Another study of 166 Finnish women with FNAIT revealed that a 1% reduction in fucosylation was linked to an 8% rise in the risk of severe bleeding (41). In mouse experiments, the removal of glycosylation from anti-HPA-1a antibodies eliminated the effector functions related to the Fc domain. Thus, deglycosylated monoclonal anti-HPA-1a antibodies, which inactivate Fc, could be employed to treat FNAIT through competitively restraining the binding of maternal alloantibodies (42).

### 2.4 Functional impacts of antibodies

#### 2.4.1 The role of anti-HPA-1a on platelets

Anti-HPA-1a antibodies can depress platelet aggregation and inhibit the adhesion of platelets to fibrinogen (43–45). Emerging evidence suggests that certain anti-HPA-1a antibodies fail to distinguish between αvβ3 and αIIbβ3, while others preferentially bind to either αvβ3 or αIIbβ3 (46, 47). Moreover, anti-HPA-1a antibodies contribute to the clearance of fetal platelets by splenic monocytes (48). In addition to the Fc-dependent mechanism, platelet antibodies can also mediate platelet clearance through Fc-independent mechanisms, including phagocytosis through Ashwell-Morell receptors in the liver (49).

C-reactive protein (CRP) has been shown to enhance the phagocytosis of platelets opsonized by anti-HPA-1a antibodies *in vitro* (50). CRP is capable of binding to C1q, thereby activating the complement cascade (51). Notably, exposure to anti-HPA-1a antibodies in serum significantly increases apoptosis of early megakaryocytes (3). Since the degree of megakaryocyte apoptosis inversely correlated with neonatal platelet counts at birth, these findings suggest that thrombocytopenia may result not only from accelerated platelet clearance but from impaired platelet production due to progenitor cell apoptosis (3). Furthermore, anti-HPA-1a antibodies can function as competitive inhibitors of the integrins, interfering with ligand binding and promoting disease progression (52, 53).

Beyond the immunological mechanisms, intrinsic platelet function in the fetus may also play a critical role in the severity of FNAIT. Although studies of healthy neonatal platelet function are limited, most indicate that neonatal platelets have compromised function. Due to their unique characteristics, fetal platelets gradually achieve hemostatic competence and functional maturation as gestation progresses (54, 55). This delicate process may be disrupted by antibody-mediated reductions in platelet count.

#### 2.4.2 Effects of anti-HPA-1a on trophoblasts

Expression of αVβ3 can be observed in placental syncytiotrophoblast cells (56). In a study analyzing placental tissues from 21 patients with FNAIT and 42 women with uncomplicated pregnancies, the incidence of inflammation in FNAIT placentas was significantly higher (57). Another investigation examined 14 placentas from FNAIT pregnancy and reported a high frequency of chronic villitis, which was alleviated by IVIg treatment (58). Additionally, FNAIT has been associated with massive chronic intervillositis (59). Experimental evidence suggests that human anti-HPA-1a mAb 26.4 can inhibit the migration and invasion of trophoblasts, implying that anti-HPA-1a antibodies may impair trophoblast functions critical for normal placentation (60). However, since these studies were conducted before the specificity of anti-HPA-1a was clarified, it remains unclear which subtypes are most strongly associated with these pathological changes. Furthermore, the deposition of complement proteins has been observed on human placental tissue exposed to anti-HPA-1a antibodies, indicating that the complement activation may contribute to impaired trophoblast function (61).

#### 2.4.3 The effect of anti-HPA-1a on endothelial cells

The epitopes of HPAs are not only found on glycoproteins present on platelets. Glycoprotein IIIa, also known as integrin β3, which contains the majority of HPAs, is expressed on the membranes of ECs and exists in a complex with integrin αV (αVβ3). When anti-HPA-1a was added to umbilical vein endothelial cells (HUVECs), it was found that the migration ability of the cells was decreased, and the integrity of their monolayers was reduced (1). In addition, anti-β3 isoantibodies generated in β3 knockout mice could cause cerebral hemorrhage in offspring through impaired angiogenesis rather than thrombocytopenia (1). It is unclear whether this discovery will contribute to the study of human ICH, since ICH occurs much less frequently in humans compared to what is observed in the animal model, and a booster effect, as depicted in mice, is not present in humans, implying that the probability of ICH does not rise with the number of pregnancies in humans (62). It appears likely that these disparities are associated with the fact that isoantibodies (in the murine model) and alloantibodies (in humans) differ in nature.

The antibody of HPA-1a can inhibit angiogenesis, promote endothelial cell apoptosis, and reduce vascular density in the brain and retina (46). Dardik et al. showed that HPA-1a antibodies obtained from mothers with severe FNAIT can induce apoptosis and notably enhance the permeability of brain ECs (63). Furthermore, the antagonists of αvβ3 are capable of influencing VEGFR2 subcellular trafficking via the endosomal system (64), and the alterations in VEGFR2 endosomal trafficking and turnover have an impact on endothelial cell function and angiogenesis (65, 66). In the FNAIT mouse model, anti-HPA-1a promoted trophoblast apoptosis by regulating uterine natural killer (uNK) cells (67).

## 3 Clinical presentation and outcome

### 3.1 Neonatal thrombocytopenia

There are a wide range of clinical manifestations in FNAIT. Firstly, asymptomatic thrombocytopenia can be identified incidentally, with no other clinical manifestations of FNAIT. The diagnosis of FNAIT needs to be considered when excluding other diseases that may cause neonatal thrombocytopenia. Secondly, newborns with FNAIT may exhibit mild bleeding symptoms, including hematoma, bruising, mild visceral bleeding, hematuria, or bloody stools. Finally, FNAIT may manifest as severe visceral bleeding, which can practically take place in the lung, bowel, or kidney (2, 16). Some infants might have thrombocytopenia without presenting visible bleeding symptoms, while platelet counts of pregnant mothers are usually normal. Accordingly, pediatricians or neonatologists should conduct a prospective evaluation and perform timely blood counts for newborns whose mothers have a previous history of FNAIT and develop skin petechiae or purple spots after birth, in order to confirm the occurrence of FNAIT. Additionally, neonatal thrombocytopenia is not uncommon and can result from various factors. Maternal underlying disorders, and newborn-specific factors such as genetic illnesses, infectious diseases, or even leukemia, may lead to neonatal thrombocytopenia.

### 3.2 Occurrence of ICH

Serious neurological complications are detected in 36% to 68% of ICH patients (16, 25), and ICH can cause 9%-46% of fetal or neonatal deaths when assessed retrospectively (68–70). A study that analyzed 21 ICH patients found a mortality rate as high as 48% (18), and about 60% of surviving newborns experienced severe neurological sequelae in the later stages (18). A study of 43 ICH cases caused by FNAIT revealed that 67% of the cases happened before 34 weeks of gestation (16). Similar outcomes were reported in a French report of ICH patients resulting from FNAIT (70). Additionally, boys were more prone to bleeding than girls (16), and the probability of recurrence for FNAIT is 79% (71). However, in FNAIT patients who did not have a baby with ICH in the previous pregnancy, the probability of having a baby with ICH in the next pregnancy is reduced to 7% (71).

### 3.3 Other pregnancy complications: lower birth weight and miscarriage

Pregnant women alloimmunized with anti-HPA-1a antibodies have a higher incidence of intrauterine growth restriction (IUGR) or a lower birth weight in their babies (72–75). A similar finding was found that pregnant women with anti-HLA class I antibodies had neonates with thrombocytopenia with decreased birth weight, and lower placental weight (76). It is speculated that the gender-nonspecific reason for the decreased birth weight might be placental inflammation, resulting from the binding of anti-HPA-1a to GPIIIa (β3) expressed on the surface of syncytiotrophoblasts (8, 77), based on studies revealing placental inflammation in FNAIT pregnancies (57, 58, 61). Moreover, anti-HPA-1a antibodies were reported to induce chronic inflammation in the placenta (78). It has been found that inflammation in the placenta, rather than systemic inflammation, determines the severity of FNAIT when detecting markers of systemic inflammation (79). Additionally, it has been suggested that complement activation in the placenta leads to fetal growth restriction in FNAIT cases (61). In a mouse model specific to HPA-1a for FNAIT, a notable influence of HPA-1a antibodies was observed on the neonatal birth weights of HPA-1a-positive pups, but not on those without HPA-1a (80). This finding supports the role of maternal anti-HPA-1a antibodies in birth weights. Moreover, boys were more likely to experience low birth weight than girls (81). Thus, male fetuses may indicate more severe neonatal outcomes (82).

Human anti-HPA-1a antibodies are associated with intrauterine death (83). When female β3-deficient mice were alloimmunized by transfusing β3-expressing platelets and then bred with wild-type males, the fetal resorption rate was significantly increased (84). Furthermore, even though the majority of FNAITs arise from αIIbβ3 mismatches, there are cases caused by alloantibodies against GPIbα (85). When GPIbα knockout female mice were mated with wild-type males, these female mice had a higher miscarriage rate than β3-deficient mice (83). Therefore, the function of anti-HPA-1a on FNAIT has been underestimated, as those cases of miscarriage are overlooked (1). Indeed, Skariah et al. (31) reported 8 women with possible FNAIT and 11 confirmed FNAIT with a history of recurrent pregnancy losses and/or implantation failures. All women successfully delivered live-born infants. Notably, none of the 8 women with possible FNAIT developed alloimmunization when managed with a protocol involving antenatal screening, serial monitoring, and immunosuppressive therapy with prednisone and IVIg (31). In addition, HPA incompatibility was associated with increased Th1 immunity and NK cell cytotoxicity, which may contribute to the elevated risk of miscarriage observed in FNAIT-affected pregnancies.

## 4 Diagnosis

### 4.1 Platelet count

The current diagnosis of FNAIT primarily depends on clinical manifestations and laboratory tests performed on newborns after birth. It is diagnosed when a newborn exhibits postnatal bleeding symptoms or the mother has a history of FNAIT in a previous child, despite her normal platelet count, and neonatal thrombocytopenia is present in peripheral blood (platelet count less than 100×10^3^/μL). Neonates with thrombocytopenia are typically detected within the initial 72 hours after birth (86). Nevertheless, for neonates with mild to moderate disease (>50×10^3^/μL) who do not have petechiae, it is often overlooked (87). Moreover, for the diagnosis, the presence of positive anti-platelet antibodies in both the newborn and the mother is necessary, along with evidence of maternal-fetal HPA system incompatibility. At the same time, other conditions that may lead to thrombocytopenia should be ruled out.

### 4.2 Antibody detection

Whenever FNAIT is suspected, it is essential to conduct anti-platelet antibody testing in the maternal blood (88, 89). A variety of approaches have been employed for testing anti-platelet antibodies, such as the monoclonal antibody-specific immobilization of platelet antigen assay (MAIPA), the platelet immunofluorescence test (PIFT), and recently developed assays using bead-based technologies, such as immune-complex capture fluorescence analysis (ICFA) and platelet antibody detection kit-Luminex xMAP (PAK Lx) (Immucor, Georgia, USA) (90). MAIPA has been regarded as the gold standard for detecting anti-platelet antibodies for many years (26). However, this approach is extremely labor-intensive and time-consuming (6–8 hours), and it must be conducted in a specialized laboratory. Conversely, ICFA only takes 2 hours to complete the testing (91), and the PAK Lx test takes about 3 hours (92). However, if weakly reactive antibodies or broadly reactive antibodies are detected, MAIPA should be used further to confirm the diagnosis. Laboratory methods with high sensitivity, such as surface plasmon resonance (SPR) assay, can detect anti-HPA antibodies of low affinity (93, 94). Furthermore, the employment of IgG receptors, specifically FcγRIIIa in this instance, on SPR chips can provide quantitative and qualitative details regarding platelet-bound anti-HPA-1a antibodies (95).

Up to 30% of multiparous women may have anti-HLA antibodies (96), which have occasionally been associated with FNAIT; however, evidence for clinical FNAIT due to anti-HLA antibodies is insufficient as a primary reason. Moreover, anti-A or anti-B antibodies have also been rarely related to FNAIT (97).

### 4.3 Platelet antigen typing

In clinical settings, samples from parents should be examined at least for HPA-1, -2, -3, -5, and -15, and in some cases, for other low-frequency antigens as well. The detection of HPA phenotype typically involves the use of antisera containing recognized antibodies for the detection of HPA, such as PIFT and MAIPA, as well as flow cytometric analysis and other approaches (98, 99). The methods for genotype determination comprise polymerase chain reaction (PCR) and gene chip technology. Recently, a new study reported the non-invasive prenatal diagnosis of fetal platelet genotype through digital droplet PCR (ddPCR) for the detection of HPA-1, -3, -5, and -15, utilizing cell-free DNA extracted from plasma (95). Furthermore, a method based on next-generation sequencing (NGS), which can precisely determine all known polymorphisms of human platelet antigens, has been reported (100). Having the capability of sequencing up to 96 samples concurrently, their HaloPlex design, which is used for NGS to enrich targets, can be applied for high-throughput HPA genotyping (100).

## 5 Antenatal management

The babies of FNAIT can be classified based on medical history. If there was a sibling with ICH or severe bleeding, the index pregnancy is defined as “high-risk”. If a sibling has experienced FNAIT but no ICH, it is considered a “standard risk.” The objective of FNAIT management is to prevent ICH during pregnancy and the perinatal period, as well as the associated neurological sequelae and mortality. Prompt diagnosis and treatment are crucial for newborns to restore normal hemostasis. In certain circumstances, FNAIT can be diagnosed during pregnancy if there is a family history of FNAIT among immediate or second-degree relatives, or if intrauterine ICH is identified through prenatal ultrasonography. Although only a small percentage of FNAIT patients develop severe thrombocytopenia and ICH, when it happens, the outcomes are grave. Hence, aggressive preventive measures should be employed. Indeed, RCTs (**Table 2**) and non-RCTs (**Table 3**) have been reported, demonstrating the impact of prenatal management on FNAIT and their outcomes.

**Table 2:** Randomized controlled trials (RCTs) on prenatal management of FNAIT.

Author (publication year)	Number of research subjects	ICH in sibling, n (%)	FBS, n (%)	IUPT, n (%)	FBS/IUPT related adverse event, n (%)	IVIg/steroids related side effects, n (%)	ICH, n (%)	Neonatal PLTs (×10^3^/μL)	Conclusions
Bussel JB, et al. (1996) (132)	IVIg 1 g/kg/wk: n=28	6 (21.4)	All	0	5 (17.9)	0	0	96	IVIg is suitable for severe thrombocytopenia in FNAIT cases, and dexamethasone does not increase the effect of IVIg.
	IVIg 1 g/kg/wk + dexamethasone: n=26	4 (15.4)	All	0	–	2 (7.7)	0	110.1	
Berkowitz RL, et al. (2006) (131)	High risk: IVIg 1 g/kg/wk: n=21	4 (19.0)	All	0	11 (13.9)	–	3 (3.8)	66.8	FBS may significantly increase fetal mortality rate.
	High risk: IVIg 1 g/kg/wk + prednisone 1 mg/kg/d: n=19	3 (15.8)	All	0		–		99.4	
	Standard risk: IVIg 1 g/kg/wk: n=19	0	All	0		–		33 (84.6) > 50	
	Standard risk: prednisone 1 mg/kg/d: n=20	0	All	0		–			
Berkowitz RL. et al. (2007) (116)	IVIg 2 g/kg/wk: n=37	0	All	0	2 (5.4)	12 (32.4)	1 (2.7)	169	Both groups of treatments have shown good results. For pregnant women who did not give birth to infants with ICH, cordocentesis is not necessary.
	IVIg 1 g/kg/wk + prednisone 0.5 mg/kg/d: n=36	0	All	0	2 (5.6)	13 (36.1)	1 (2.8)	134	
Paridaans NP. et al. (2015) (115)	IVIg 0.5 g/kg/wk: n=12	0	0	0	–	0	0	81	They recommended treating standard-risk patients with 0.5 g/kg/wk IVIg.
	IVIg 1 g/kg/wk: n=11	0	0	0	–	0	0	110	
Kamphuis M, et al. (2016) (114)	IVIg 0.5 g/kg/wk: n=46	0	0	0	0	–	0	112	For FNAIT pregnant women who did not give birth to ICH neonates, 0.5 or 1 g/kg/wk IVIg has a similar therapeutic effect.
	IVIg 1 g/kg/wk: n=63		0	0	0	–	0	119	

ICH, intracranial hemorrhage; FBS, fetal blood sampling; IUPT, intrauterine platelet transfusion; IVIg, intravenous immunoglobulin; “-”, not known or not applicable.

**Table 3:** Non-randomized controlled trials (non-RCTs) on prenatal management in FNAIT.

Author (publication year)	Type of study	Number of research subjects	ICH in sibling, n (%)	FBS, n (%)	IUPT, n (%)	FBS/IUPT related adverse event, n (%)	IVIg/steroids related side effects, n (%)	ICH, n (%)	Neonatal PLTs (×10^3^/μL)	Conclusions
Murphy MF, et al. (1994) (169)	Retrospective	IVIg + IUPT ± steroids: n=8	6 (75)	All	All	1 (12.5)	–	1 (12.5) (before therapy)	340 (after IUPT)	Non-invasive treatment is effective for pregnant women with FNAIT, and there are no adverse consequences of invasive treatment.
		IUPT ± steroids: n=7	5 (71.4)	All	All	0	–	2 (28.6) (before therapy)	305 (after IUPT)	
Kornfeld I, et al. (1996) (170)	Retrospective	IVIg + IUPT: n=4	1 (25)	All	All	1 (25)	0	0	182	IVIg seems to improve the outcomes of infants at risk of FNAIT.
		IVIg: n=6	1 (16.7)	All	0	1 (16.7)	0	0	98	
Kaplan C, et al. (1998) (171)	Retrospective	IVIg: n=27	7 (25.9)	All	1 (3.7)	–	–	2 (7.4)	69	Fetal platelet count must be tested twice during pregnancy to evaluate the effectiveness of the treatment.
		Steroids: n=10	–	All	–	–	–	–	–	
Sainio S, et al. (1999) (103)	Retrospective	IVIg ± IUPT: n=11	1 (9.1)	All	9 (81.8)	2 (18.2)	1 (9.1)	0	109	IVIg or platelet transfusion to the fetus is not sufficient to protect severely affected fetuses from FNAIT.
		IUPT: n=4	0	All	All	2 (50)	–	0	76	
Silver RM, et al. (2000) (172)	Prospective	IVIg to the mother: n=8	3 (30)	All	–	2 (20)	–	0	–	The inconsistent effect of IVIg on increasing fetal platelet count may be due to maternal or placental factors, rather than the inhibitory effect of IVIg on fetal platelet destruction.
		IVIg to the fetus: n=2		All	–		–	0	–	
Tiblad E, et al. (2003) (173)	Retrospective	IVIg (one had prednisolone): n=9	5 (55.6)	0	0	–	–	0	90	Non-invasive treatment can be applied to FNAIT cases.
		IUPT: n=3	0	All	All	All	–	0	47	
		No treatment: n=6	2 (33.3)	0	0	–		1 (16.7)	9	
Birchall JE, et al. (2003) (174)	Retrospective	IVIg ± IUPT: n=18	6 (33.3)	All	6 (33.3)	3 (16.7)	1 (5.6)	1 (5.6) (before therapy)	81	FNAIT cases should be stratified based on medical history.
		IUPT: n=31	11 (35.5)	All	All	10 (32.3)	–	2 (6.5) (before therapy)	–	
		FBS ± IUPT: n=7	0	All	5 (71.4)	2 (28.6)	–	0	–	
Radder CM, et al. (2004) (108)	Prospective	IVIg ± IUPT: n=37	8 (16.0)	26 (70.3)	26 (70.3)	0	–	0	67	IVIg treatment during pregnancy did not affect the development of the children’s nervous system.
		FBS ± IUPT: n=13		All	9 (69.2)	2 (22.2)	–	2 (15.4)	32	
Kanhai HH, et al. (2006) (175)	Prospective	IVIg 1 g/kg/wk: n=7	All	3 (42.9)	3 (42.9)	–	–	0	39, 14, 15, and 10 for four patients	For severe FNAIT cases and those who have risk factors for cordocentesis, IVIg can be chosen.
Bertrand G, et al. (2006) (98)	Prospective	IUPT: n=2	0	All	All	–	–	0	210	The concentration of anti-HPA-1a antibody can be used to guide invasive management.
		IVIg: n=4	0	2 (50)	2 (50)	–	–	0	204	
		IVIg + corticoids: n=13	2 (15.4)	1 (7.7)	1 (7.7)	–	–	0	118	
Yinon Y, et al. (2006) (176)	Retrospective	IVIg: n=24	0	7 (29.2)	0	–	–	0	118	For FNAIT fetuses, IVIg treatment is effective and safe.
		No treatment: n=6	0	0	–	–	–	0	24	
te Pas AB, et al. (2007) (158)	Retrospective	IVIg: n=13	5 (38.5)	–	2 (15.4)	–	–	0	83	They do not recommend IVIg treatment for FNAIT cases.
van den Akker ES, et al. (2007) (177)	Retrospective	IVIg: n=53	5 (9.4)	0	0	–	–	0	125	Non-invasive treatment for FNAIT is effective and safe.
		FBS + IVIg: n=33	11 (33.3)	All	–	3 (9.1)	–	0	174	
		FBS + IUPT: n=13	0	All	All	0	–	0	145	
Ghevaert C, et al. (2007) (143)	Retrospective	IUPT ± IVIg ± corticosteroids: n=40	–	All	All	3 (7.5)	–	4 (10)	107	There is a relatively high risk of injecting platelets into the fetus.
		IVIg and/or corticosteroids: n=7	–	–	0	1 (14.3)	–	0	7-219	
		No treatment: n=8	–	–	–	–	–	0	6-84	
Giers G, et al. (2010) (178)	Retrospective	IVIg to the fetus + IUPT: n=10	–	All	All	0	–	0	189 (for the fetus)	After weekly administration of IVIg, fetal IgG levels increased, but fetal platelet count did not change.
Bertrand G, et al. (2011) (86)	Retrospective	IVIg 1 g/kg/wk: n=27	9 (9.8)	0	0	–	–	0	89	The concentration of maternal anti-HPA-1a antibody before 28 weeks can predict fetal status.
		IVIg 1 g/kg/wk + prednisone 0.5 mg/kg/d: n=54		0	0	–	–	0	135	
		Prednisone 0.5 mg/kg/d: n=11		0	0	–		0	46	
Mechoulan A, et al. (2011) (179)	Retrospective	IVIg: n=17	5 (29.4)	9 (52.9)	0	1 (11.1)	–	0	68	IVIg treatment achieved good results.
		IVIg + corticosteroids: n=6	0	0	0	–	–	0	78	
Bussel JB, et al. (2010) (13)	Retrospective	IVIg 1 g/kg/wk: n=5	All	All	1 (20)	3 (60)	–	1 (20)	165	Patients should be stratified according to the occurrence of ICH in siblings.
		IVIg 1 g/kg/wk + prednisone 1 mg/kg/d: n=19	All	All	0	0	–	3 (15.8)	85	
		IVIg 2 g/kg/wk: n=4	All	All	0	0	–	0	112	
		IVIg 2 g/kg/wk + prednisone 1 mg/kg/d: n=9	All	All	0	0	–	1 (11.1)	135	
Van Der Lugt NM, et al. (2015) (14)	Retrospective	IVIg 1 g/kg/wk: n=5	2 (40)	0	0	–	–	1 (20)	63	Non-invasive treatment is effective for pregnant women with a history of FNAIT.
		IVIg 0.5 g/kg/wk: n=17	0	0	0	–	–	0	104	
Lakkaraja M, et al. (2016) (104)	Prospective	IVIg 2 g/kg/wk: n=51	0	42 (82.4)	17 (33.3)	4 (7.8)	The most common: headache 11 (21.6)	3 (3)	169	The two recommended treatment options can effectively increase fetal platelet count and are very safe. It may be considered to cancel invasive examinations.
		IVIg 1 g/kg/wk + prednisone 0.5 mg/kg/d: n=49	0	38 (77.6)	20 (40.8)	6 (12.2)	The most common: headache 10 (20.4)		135.3	
Wienzek-Lischka S, et al. (2020) (110)	Retrospective	IVIg: n=12	–	0	0	–	The most common: headache 11 (91.7)	1 (8.3)	187	IVIg treatment is effective, and invasive treatments should be abandoned.
Ernstsen SL, et al. (2022) (111)	Retrospective	IVIg: n=403	90 (22.3)	–	–	–	–	7 (1.7)	–	For low-risk pregnant women, no treatment did not increase the risk of fetal/neonatal ICH.
		No treatment: n=71	7 (9.9)	0	0	0	–	2 (2.8)	–	

ICH, intracranial hemorrhage; FBS, fetal blood sampling; IUPT, intrauterine platelet transfusion; IVIg, intravenous immunoglobulin; “-”, not known or not applicable.

### 5.1 Fetal blood sampling and intrauterine platelet transfusions

Approximately 40 years ago, FBS and IUPTs were the primary treatment options for reducing the incidence of ICH (101). However, the transfused platelets have a short half-life, necessitating weekly transfusions. The pre-transfusion platelet counts are frequently much lower than 50×10^3^/μL merely seven days after transfusion, suggesting that weekly transfusions may not be sufficient to sustain the ideal platelet count (102). A systematic review of complications caused by FBS or IUPTs revealed that the incidence of complications was 11%, with emergency cesarean section being the most common (20). Fetal bradycardia is frequently observed, which may be attributed to the larger plasma volume transfused, and in a fetus with thrombocytopenia, the risk of bleeding is high (103). Additionally, invasive fetal diagnosis, such as FBS, chorionic villous sampling, or amniocentesis, not only carries procedure-related risks but may also enhance the alloantibody production for the mother (20, 104). Considering the short lifespan of transfused platelets, regular transfusions are necessary, which raises the overall risk of fetal loss (105). Consequently, if FBS is taken into account, it ought to be carried out only by invasive fetal medicine experts. It is noteworthy that a recent study indicated that invasive treatment is not necessary if IVIg treatment is administered (104).

### 5.2 The use of IVIg

#### 5.2.1 The impact of IVIg

At present, in most Western countries, high-dose IVIg treatment is often considered for pregnant women with HPA-1a alloimmunization to prevent ICH of the fetus. This off-label treatment is commonly considered effective, but it is also costly. Since IVIg has been used for this condition for several decades, it is often considered unnecessary to investigate the efficacy of IVIg in a placebo-controlled clinical study (106). Consequently, solid evidence is lacking regarding the efficacy of IVIg in preventing severe FNAIT (20, 106).

The treatment of IVIg has been a core component of clinical guidelines (90, 107). The following working mechanisms have been suggested: Firstly, IVIg may dilute and reduce the concentration of alloantibodies in the mother. Secondly, IVIg may competitively bind to the neonatal Fc receptor, thus reducing the quantity of antibodies that enter the fetus. Lastly, the competitive binding may occur in the fetal spleen, leading to reduced platelet destruction in the spleen (108, 109).

Besides, IVIg treatment has been reported to suppress placental inflammation. Seven women who underwent IVIg administration showed no indications of chronic villitis, whereas six untreated pregnancies showed it (58). For pregnant women who have a history of FNAIT, the application of IVIg led to a favorable outcome for all newborns (110). A systematic review, which analyzed 315 pregnancies treated solely with IVIg, found an effective rate of 98.7% in preventing ICH (20). This is in line with the rate of 97.3% reported in the Cochrane analysis (106), which encompassed 37 pregnancies treated solely with IVIg. The latest international guideline has considered IVIg as the first-line treatment for FNAIT (107).

**Figure 2:**
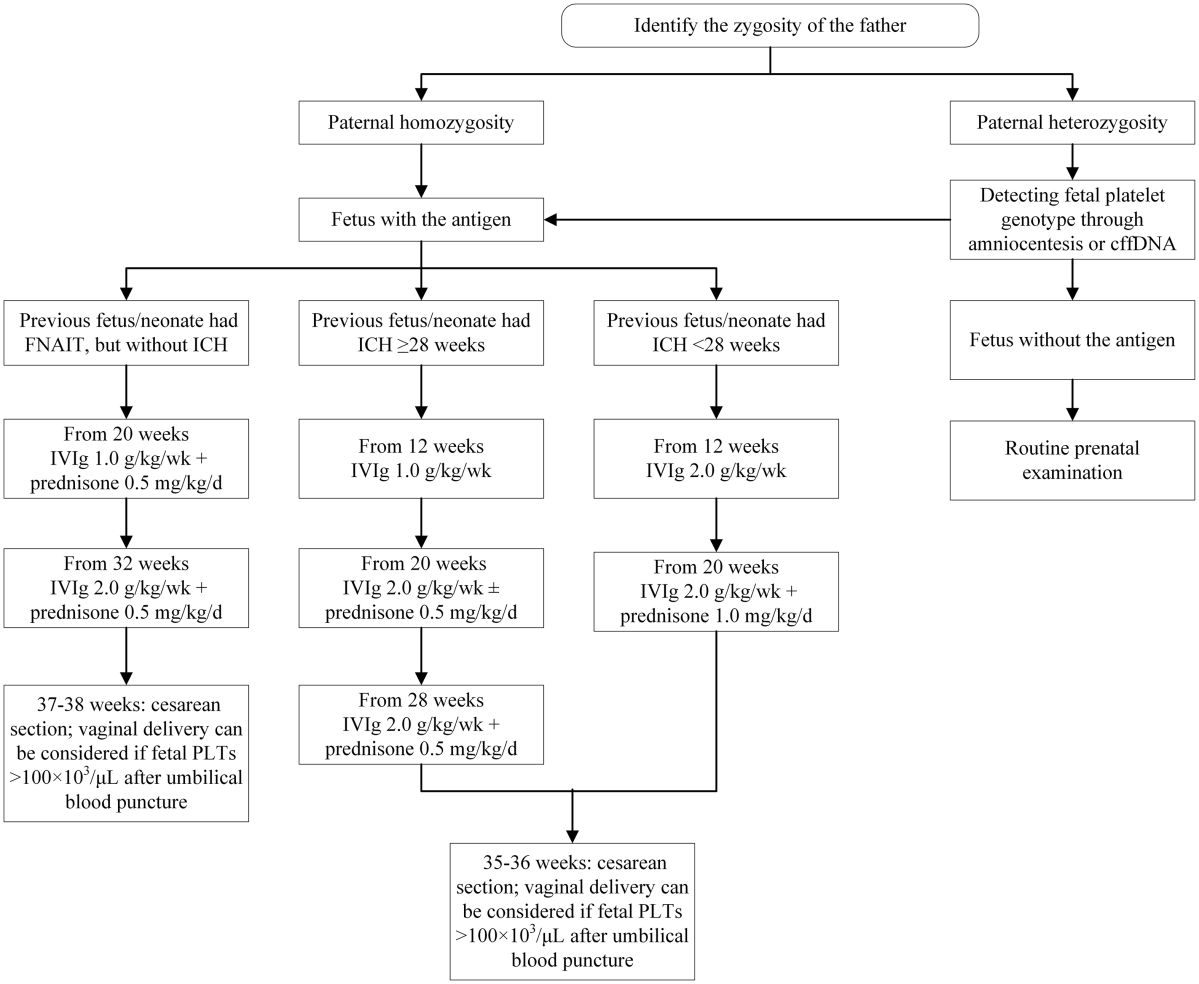
The treatment flow for pregnant women with a history of FNAIT. cffDNA, cell-free fetal DNA; ICH, intracranial hemorrhage; IVIg, intravenous immunoglobulins; PLTs, platelets.

It should be noted that a recent report involving 64 pregnant women with FNAIT resulting from HPA-1a incompatibility suggested that if the first-affected baby did not have an ICH, ICH would not occur in the second pregnancy: 0 out of 64 in such cases (111). They found no evidence indicating that omitting antenatal IVIg treatment in low-risk pregnancies raises the risk of neonatal ICH (111). However, the sample size in this study is relatively small, further research with a larger sample size is needed. Withholding immunoglobulin treatment may pose potential risks to these low-risk pregnant women. The treatment flow in the United States for pregnant women with a history of FNAIT is shown in **Figure 2** (112).

#### 5.2.2 The dose of IVIg

For high-risk patients, a dose of 1–2 g/kg/wk is recommended (20), while for standard-risk patients, a dose of 0.5 g/kg/wk is often considered (113, 114), which has a comparable therapeutic effect compared to 1 g/kg/week (14, 115). In contrast, higher dose treatment (for instance, 2 g/kg/week) was unclear to draw a definitive conclusion due to a lack of sufficient controls for comparison (13, 116). The initiation time of IVIg also varies. In Europe, 28 weeks is typically regarded as the starting point, while in the US, treatment usually commences at 20 or 24 weeks (56). The latest guidelines recommend treating high-risk patients from 16 weeks of pregnancy and standard-risk patients from 20–22 weeks (107). The duration of treatment typically spans the entire pregnancy period.

#### 5.2.3 The side effects of IVIg

As a blood product, IVIg may transfer infectious diseases, such as hepatitis C, which was reported in 1995 (117). Thereafter, manufacturers have improved the production process, including the adoption of virus inactivation technology and nanofiltration (118). Currently, IVIg treatment is considered safe and well-tolerated, with only mild side effects in the majority of patients (119–121). Side effects, such as fever and headache, may be related to higher dosages (119). Furthermore, IgA-related anaphylaxis has been reported in a few patients with IgA deficiency (118). Patients with underlying kidney disease may experience additional kidney problems during treatment (122, 123). Moreover, high-dose IVIg treatment may lead to aseptic meningitis (118). IVIg contains isohemagglutinins (anti-A and anti-B). Hence, IVIg infusion can lead to coating red blood cells (RBCs) with isohemagglutinins, resulting in either clearance by the reticuloendothelial system, intravascular hemolysis, or both (124–126). Women who are not blood group O (A and B) have a higher occurrence of hemolysis resulting from IVIg (124, 127, 128). Therefore, patients undergoing IVIg treatment should be closely monitored upon initial exposure.

In an analysis of 20 cord blood samples, no significant alterations in the immune system were observed following *in-utero* exposure to IVIg treatment. A subsequent questionnaire study revealed that the incidence of pediatric infectious diseases was not significantly increased (129). Ward et al. (130) reported better neurodevelopmental outcomes in 71 children who had received antenatal IVIg compared to 71 untreated siblings. However, this study relied largely on telephone surveys and was limited by a substantial loss-to-follow-up rate (37%), thereby reducing the strength of its conclusions. Consequently, large-scale, long-term prospective studies are needed to clarify the impact of antenatal IVIg exposure.

### 5.3 Advantage of adding corticosteroids to IVIg

The advantage of incorporating corticosteroids into IVIg remains uncertain. Several studies discovered an increase in platelet counts; platelet count exceeding 25×10^3^/μL at the second sampling, an increase of platelet count more than 10×10^3^/μL compared to the first sampling, or a platelet count higher than 40×10^3^/μL that did not decrease by more than 10×10^3^/μL (106, 131). Studies that compared treatments using IVIg to IVIg combined with steroids demonstrated no significant differences in platelet count, ICH, or mortality (116, 132). A systematic review published in 2017 found that adding glucocorticoids was not beneficial (20). Furthermore, dexamethasone is not advised because high doses may cause oligohydramnios (107), while low doses have no effect (132). Therefore, it is often recommended to use prednisone; however, the potential benefits need to be carefully evaluated (106) since corticosteroids are linked to hypertension and diabetes, and both of which can impact the quality of life for patients (133).

### 5.4 Anti-neonatal Fc receptor therapy

The blockage of the neonatal FcRn is considered the principal mechanism underlying the therapeutic effect of IVIg. Consequently, blocking the receptor with anti-FcRn antibodies in IVIg offers the potential to replicate the effect of anti-FcRn monoclonal antibodies (mAbs), which can reproduce this effect while avoiding the adverse events associated with high-dose IVIg. Clinical studies have demonstrated that anti-FcRn mAbs can increase platelet counts, reduce the miscarriage rate, and decrease bleeding tendencies in animal models (1, 83, 134). Notably, their effective dose is over 200-fold lower than that of IVIg, highlighting their potential for greater efficacy and improved safety (134). Furthermore, unlike IVIg, anti-FcRn antibodies are not derived from human plasma and therefore carry no risk of transmitting infectious diseases. Their production cost is also comparatively lower. While these agents show considerable promise, their safety and efficacy still require further validation in clinical trials. In particular, concerns remain that a complete blockage of transplacental IgG transport could result in unintended and clinically significant adverse effects (26).

### 5.5 Antigen-specific therapy

Currently, several antigen-specific therapeutic strategies are under investigation for the treatment of FNAIT. One approach involves the use of mAbs that neutralize or silence the pathogenic effect of maternal alloantibodies on fetal platelets (135). Another strategy is to design agents targeting B cells responsible for alloantibody production (136). More recently, Chimeric Autoantibody Receptor (CAAR) T cell therapy has emerged as a promising option, as it can selectively eliminate autoreactive or alloantibody-producing B cells while sparing protective immune functions (137). This targeted approach may ultimately prove effective in managing alloimmune response against HPA-1a.

## 6 Preimplantation genetic diagnosis and *in vitro* fertilization

Genetic screening can be performed before transplantation to identify and implant embryos with the HPA-1bb genotype, thereby preventing the development of FNAIT (138). This approach may also be considered when selecting HPA-matched sperm donors. In 2014, the first successful case was reported in Norway, where a woman gave birth to a healthy child with normal platelet counts after undergoing this strategy (139). However, when the mother is HPA-1bb and the father is HPA-1aa, preimplantation genetic testing is unnecessary, as the offspring will inherit the HPA-1ab genotype obligatorily.

## 7 Mode and timing of delivery

It remains unclear whether caesarean section reduces the risk of ICH in neonates affected by FNAIT (106, 140). The 2019 guidelines emphasized that no controlled studies have directly compared delivery methods in this context (107). When the fetal platelet count exceeds 50×10^3^/μL, vaginal delivery may be considered, although this requires confirmation of platelet levels through FBS prior to labor (132). In a study of pregnancies with standard risk for FNAIT, no increased risk of ICH was observed with vaginal delivery (141). Importantly, maternal antibody concentration has been proposed as a key risk factor (86, 142). High titers (≥28 IU/mL) were associated with a greater likelihood of severe FNAIT, whereas low titers (<28 IU/mL) supported the safety of vaginal delivery (86). For cases managed by vaginal delivery, the use of instrumental assistance by using forceps and invasive monitoring, such as scalp electrodes, should be avoided to minimize bleeding risk.

In a retrospective study of 200 cases of FNAIT, 17 neonates were diagnosed with postnatal ICHs within the first 24 hours after delivery, suggesting that birth trauma may be a major contributing factor (143). However, the ultrasound examinations were not performed within 24 hours prior to delivery; it remains unclear whether these ICHs were antepartum or intrapartum in origin. Despite this uncertainty, it is widely recognized that high-risk FNAIT pregnancies should be delivered by cesarean section (144). Moreover, a planned delivery facilitates the timely preparation of HPA-Ag-negative platelets for immediate neonatal transfusion, thereby improving postnatal management (33).

## 8 Postnatal management of the newborn

### 8.1 Platelet transfusion

#### 8.1.1 Transfusion threshold

Neonates affected by FNAIT should undergo thorough physical examination, platelet count evaluation, and cranial ultrasound immediately after birth (145). At present, there are no established strategies to reliably prevent neonatal ICH after delivery reliably (21). Current expert consensus recommends platelet transfusion within the first week postpartum for premature infants with platelet counts below 50×10^3^/μL (146–149). The Platelets for Neonatal Transfusion-Study 2 (PlaNeT-2) trial evaluated transfusion threshold in preterm infants (<34 weeks of gestation) (150, 151). Results showed that maintaining a higher transfusion threshold (50×10^3^/μL) did not improve outcomes compared with a lower threshold (25×10^3^/μL). On the contrary, infants in the higher threshold group had a significantly increased risk of death or major bleeding (150). Consistent with this, most neonates with platelet counts above 30×10^3^/μL did not require treatment (151), except when premature or clinically unstable (107). Importantly, evidence suggests that platelet transfusion itself may increase bleeding risk in premature infants with thrombocytopenia (152). This has led to the inference that prophylactic platelet transfusion in FNAIT neonates, particularly preterm infants, may exacerbate vascular fragility and endothelial cell damage, potentially causing harm rather than benefit.

#### 8.1.2 The type of platelet input

The HPA-compatible platelets, particularly those negative for HPA-1a and HPA-5b, are considered the treatment of choice for transfusion in FNAIT neonates, as they can achieve a platelet count increase in approximately 95% of cases and significantly improve survival (56). Unfortunately, only a limited number of hospitals are able to provide such emergency HPA-matched platelet transfusions (153). When compatible platelets are unavailable, maternal platelets can serve as an alternative. In such cases, platelets are collected, washed, irradiated, and leukoreduced prior to transfusion. As this process requires several hours, antenatal preparation, including maternal platelet apheresis before delivery in high-risk pregnancies, is recommended. As an interim measure, random donor platelets may be used (154). In a national cohort study of 102 first-diagnosed FNAIT cases, similar short-term platelet increments were observed with both HPA-matched and random donor platelets (155). Nevertheless, random platelet transfusion should be regarded as a temporary solution until HPA-compatible platelets are secured (156, 157).

### 8.2 The therapy of IVIg

The infusion of IVIg administered within 1–3 days after birth may be used as a supplementary therapy alongside platelet transfusion, depending on the neonate’s response (148, 158). Because the onset of IVIg action requires approximately 20 hours, it is generally regarded as an adjunctive rather than primary treatment. IVIg can help raise platelet counts and improve hemostasis, particularly when antigen-negative platelets are unavailable. However, unlike its antenatal use, IVIg is not recommended as the sole postpartum therapy, as its effects are slower than the immediate benefit of platelet transfusion (158).

## 9 A prophylactic strategy for FNAIT

A Norwegian monitoring study demonstrated that approximately 75% of HPA-1a alloimmunization events occur after childbirth, highlighting delivery as a major triggering factor for the maternal immune response (112). Other studies have reported that the risk of maternal immunizations during a first pregnancy ranges from 7.9% (159) to 25% (160). Based on this, if an HPA-1a-negative mother delivers an HPA-1a-positive infant, the administration of anti-HPA-1a antibody post-partum could potentially prevent the development of HPA-1a-induced FNAIT. The European PROFNAIT consortium is actively pursuing this strategy to establish a prophylactic therapy using plasma-derived anti-HPA-1a antibodies (NAITgam). Additionally, experimental approaches aim to prevent HPA-1a immunization by modulating antigen-specific T cell responses. However, the exact contribution of T-cell reactivity to anti-HPA-1a antibody production remains poorly defined. Promisingly, oral peptide-induced immune tolerance, mediated through the expansion of tolerogenic regulatory T cells, may represent a novel prophylactic pathway (161).

## 10 Universal screening

The decision on whether to conduct FNAIT monitoring nationwide remains a controversial topic (33, 140, 162–164). Data from Norway have shown that screening can improve pregnancy outcomes (33) and be cost-effective (165). Other studies have also reached similar conclusions (140, 159). If antenatal screening is not conducted, only a small number of cases can be detected (87). In a prospective study involving 176,084 low-risk pregnancies, it was determined that screening all pregnancies in combination with antenatal treatment could decrease the mortality of FNAIT (140). However, no RCT has yet confirmed whether screening can improve clinical outcomes, and its economic benefits remain unclear.

In the United States, about 28% of HPA-1a-negative women carry the DRB3^*^0101 HLA allele, and its absence is associated with a markedly lower risk of producing high-titer anti-HPA-1a antibodies (166, 167). Thus, HLA-DRB3^*^0101 genotyping may help identify women at high risk. In addition, a prospective study showed that elevated sFlt-1 levels and an increased sFlt-1/PIGF ratio in the third trimester were significantly associated with HPA-1A alloimmunization, suggesting their value as biomarkers for risk stratification (168).

## 11 Recommendations and future directions

Despite ongoing progress, major knowledge gaps remain in understanding the pathogenesis of FNAIT, which contributes to the absence of consensus on managing at-risk pregnancies. Emerging evidence that the placenta is a direct target of maternal anti-HPA-1a antibodies, together with the observed association with increased miscarriage rates, underscores the need for further investigation. Prophylactic strategies, such as “NAITgam” are still in early development, and the implementation of universal screening and prevention programs will likely require years of additional research. Nonetheless, preliminary data suggest that population-based screening could substantially reduce severe outcomes and ease the burden on affected families.

## 12 Conclusion

This review highlights recent advances but is limited by the inclusion of only English-language studies and variability in study quality. At present, IVIg, with or without corticosteroids, remains the standard of care for high-risk pregnancies, while animal studies continue to provide promising insights for future prophylactic and therapeutic approaches. We advocate for the consideration of national screening initiatives to help prevent severe complications and mitigate the long-term socioeconomic and healthcare burden of FNAIT.
